# Cerebral Blood Flow Measurement in Healthy Children and Children Suffering Severe Traumatic Brain Injury—What Do We Know?

**DOI:** 10.3389/fneur.2020.00274

**Published:** 2020-04-16

**Authors:** Elham Rostami, Pelle Nilsson, Per Enblad

**Affiliations:** ^1^Section of Neurosurgery, Department of Neuroscience, Uppsala University, Uppsala, Sweden; ^2^Department of Neuroscience, Karolinska Institute, Stockholm, Sweden

**Keywords:** pediatric brain injury, cerebral blood flow, cerebral autoregulation, traumatic brain injury, Xenon-CT, TCD, head injury children

## Abstract

Traumatic brain injury is the leading cause of death in children. Children with severe TBI are in need of neurointensive care where the goal is to prevent secondary brain injury by avoiding secondary insults. Monitoring of cerebral blood flow (CBF) and autoregulation in the injured brain is crucial. However, there are limited studies performed in children to investigate this. Current studies report on age dependent increase in CBF with narrow age range. Low initial CBF following TBI has been correlated to poor outcome and may be more prevalent than hyperemia as previously suggested. Impaired cerebral pressure autoregulation is also detected and correlated with poor outcome but it remains to be elucidated if there is a causal relationship. Current studies are few and mainly based on small number of patients between the age of 0–18 years. Considering the changes of CBF and cerebral pressure autoregulation with increasing age, larger studies with more narrow age ranges and multimodality monitoring are required in order to generate data that can optimize the therapy and clinical management of children suffering TBI.

## Introduction

Traumatic brain injury is a leading cause of death among children and affects children all over the world. The worldwide annual incidence of pediatric TBI ranges between 47 and 280 per 100,000 children depending on the country (1). The U.S. Centers for Disease Prevention and Control estimated that in 2014, 837,000 TBI-related emergency department (ED) visits, hospitalizations, and deaths occurred among children (2).

Although the majority of TBIs are mild, a large number of children with severe TBI require neurointensive care with neuromonitoring. Despite the size of this patient population there is still a lack of evidence-based guidelines for how to manage TBI in children (3).

Acute TBI is characterized by primary and secondary injuries. Primary brain injury is the direct injury to the brain parenchyma at the time of the initial impact. The primary injury in children is even more heterogeneous and complex than in adults considering the impact of inflicted TBI. A common consequence of different primary injuries is triggering of secondary processes leading to secondary injury such as breakdown of the blood–brain barrier, neurons damage, disturbed cerebral pressure autoregulation, ischemia, and brain herniation (4, 5).

The goal of TBI management in the acute setting is to prevent secondary insults and brain injuries by achieving adequate CBF (6). This is mainly achieved by monitoring of ICP and CPP to optimize CBF, which is crucial in order to deliver substrates to the injured brain. The normal brain represents 2% of body weight but receives 15% of cardiac output. It has a high energy demand utilizing 20% of available oxygen and 60% of glucose by the whole body in the resting state. The brain glucose consumption peaks at age of 5 and is 2 times more the daily glucose use of the adult brain (7). Boys have higher brain glucose consumption compared to girls that becomes equal in adulthood (8).

The CBF to match the high demand of brain is on average 50 ml/100 g/min, higher in gray matter compared to white, 80 and 20 ml/100 g/min, respectively.

A tight regulation of blood flow and oxygen delivery is critical for neuronal survival which becomes even more important in the injured brain.

This regulation is controlled by several homeostatic mechanisms. The most important ones are cerebral metabolism, cerebral pressure autoregulation, PaCO_2_ and PaO_2_.

CBF is tightly coupled to cerebral metabolism and during normal conditions CBF is well-correlated with cerebral metabolic rate of oxygen (CMRO_2_). The CMRO_2_ is 3.5 ml/100 g/min and is four times higher in gray matter compared to white.

Cerebral pressure autoregulation is the mechanism that regulates this constant cerebral blood supply in spite of fluctuations in blood pressure by adjusting the diameter of cerebral vessels. An increase in MAP/CPP will lead to vasoconstriction and a decrease will lead to vasodilatation. This protects the brain from brain ischemia and edema/hemorrhage (9).

In the seminal work by Lassen it was shown that the CBF is maintained constant at changes in mean arterial pressure (MAP) between 60 and 160 mm Hg in healthy adults and the classic triphasic curve of CBF was presented (10). However, this range has shown to be narrower in infants. The upper limit of MAP was shown to be as low as 45 mmHg at birth and increased to 100 during first weeks after birth (11). In another paper, the lower limit was reported to be within the same range as in adults (46–70 mmHg) and no correlation with age could be seen (12).

PaCO_2_ is the most potent vasodilatory agent that increases the CBF by 2–4%/mmHg. The CBF response to PaO_2_ changes occur in seconds and is used clinically by hyperventilation in order to decrease ICP.

The influence of PaO_2_ on cerebral circulation is much less compared to PaCO_2_ and with less clinical significance. The changes are minimal in PaO_2_ above 50 mmHg and when PaO_2_ is below 50 mmHg the CBF increases but the changes are slow and take more than 6 min.

Although the relationships between ICP, MAP, CPP, and CBF can be straightforward in the healthy brain it is more complex in the injured brain in particular in the developing brain. Many physiological parameters such as CO_2_ reactivity, O_2_ reactivity, and cerebral pressure autoregulation can be disturbed and compromise the need of adequate substrate delivery. Disturbed cerebral pressure autoregulation and hypoperfusion have been observed in children with TBI and has been correlated to worse outcome (13–16). Younger children <4 years are at a higher risk of impaired cerebral pressure autoregulation and have worse outcome than older children.

Although a lot of work has been done in order to monitor and understand the cerebral hemodynamic changes following TBI there is little known about children with TBI. In order to optimize therapeutic and management protocols of children with severe TBI an extensive understanding of the normal physiology and cerebral hemodynamic changes following TBI is necessary. In this review we have made an effort to summarize current studies on monitoring of CBF in healthy and severely brain injured children.

### CBF in Healthy Pediatric Population

One of the earliest studies on healthy children was performed by Settergren et al. who measured CBF in 70 healthy, anesthetized children between 11 days and 15 years of age. The children had slightly higher CBF compared to adults and 77% had mean CBF below 65 ml/100 g/min (17).

The gradual changes of CBF by age have been reported in smaller studies. Using PET in 24 healthy children it was shown that regional CBF and CMRO_2_ were low in neonates but increased in children >1 year and peaked at 3–8 years to reach adult values at >8 years (18). The values in this study are given as ratio compared to adults thus comparison of absolute CBF values with other studies is difficult. Although, this study reported on normal values, the included children had underlying diagnoses such as Moyamoya disease, skull deformity and craniosynostosis, but they had normal development and no signs of intracranial hypertension. Several of these diagnoses may be associated with disturbed CBF such as Moyamoya disease. In a previous study using133Xe intravenous injection method it was shown that children with non-symptomatic Moyamoya disease have lower CBF than normal children so it has been questioned if reported CBF levels should be interpreted as normal (19). There is also a difference in the CBF alteration between white and gray matter where CBF decreases in gray matter with age but not in white matter (20). The peak of CBF in gray matter was detected at the age of 3–4 years (20).

The gradual changes in CBF by age have also been demonstrated indirectly by transcranial doppler. Verlhac who used transcranial Doppler (TCD) found that the CBF velocity in MCA increased with age starting in newborns at around 24 cm/s and peaking in 6–10 years of age to 97 cm/s. This was followed by a decrease in older children (age 10–16.9 years) to 81 cm/s (21). In healthy adults it has been shown that CBF velocity is decreased to 50 cm/s (21, 22). It should be noted that TCD measures changes in flow velocity in one large vessel usually MCA and is not a direct measurement of global or regional CBF.

The dynamic changes in CBF through childhood was also shown by Chiron et al. (23) using SPECT and 133Xe in 42 children. The mean CBF at birth was found to be 50 ml/100 g/min and peaked by the age of 5–71 ml/100 g/min.

Using perfusion CT in 77 children aged 7 months to 18 years, Wintermark et al. found values of 40 ml/100 g/min at 6 months with a peak of 130 ml/100 g/min at 2–4 years of age that stabilized at 50 ml/100 g/min at 7–8 years of age (24). The study included healthy children based on normal CT-scans but the children were admitted for headache and mild TBI which could affect the CBF measurements in children as shown in other studies (25).

Recent advancement in imaging techniques provides the opportunity to use non-invasive and radiation free CBF measurements. Carsin-Vu et al. used MRI—arterial spin label (ASL) technique in 84 children with headache, seizure and autism. The children were aged 6 months up to 15 years and the mean CBF measured was 64 ml/100 g/min in gray matter and 29.3 ml/100 g/min in white matter (26).

Most of the studies reporting on normal CBF values have been performed on children with an underlying diagnosis and in a small number of subjects in different age ranges. Paniukov et al. have recently performed a study on healthy children using MRI-ASL in 96 volunteers and they also found an age-dependent increase in CBF. The CBF increased by 3.2 ml/100 g/min per year from 2 to 7 years with no difference in males and females (27). Most of the studies mentioned above could not show any differences in CBF between boys and girls. Both boys and girls show linear correlation between CBF and age with a shifting point around puberty (28). However, in the largest CBF study, including 922 youths aged 8–22, Satterthwaite et al. reported that CBF continue to decline after puberty in males but increased thereafter in females (29). This indicates that the age when TBI occurs may have different effects in females compared to males.

Several of the studies above also reported on regional variation in CBF that corresponds well with brain maturation and development that may have different impact in the functional outcome depending on the age of children.

One can conclude that there is a great variation in CBF in children related to age and that the values are very different compared to adults. Thus, when evaluating CBF measurements in children it is important to take into account the large difference observed between children and adults and also the large differences between age groups within the pediatric population. Accordingly, narrow age ranges should be used when normal CBF reference values are presented. However, one should keep in mind some limitations of these studies such as that different methods for CBF measurements were used and not all of them report on absolute CBF values which limits a comparison between the studies (Table 1). There is also a large variation in age span studied and most of the studies have few cases. Furthermore, most of the included subjects considered as healthy were actually either admitted for different symptoms or had an underlying diagnosis. Thus, further studies are needed in particular in younger healthy children to obtain absolute CBF values.

**Table 1:** Summary of papers reporting on CBF measurements in healthy children.

**References**	**Method**	***n***	**Age**	**CBF (ml/100 g/min)**	**Age of peak CBF**	**Subjects**
Settergren et al. (17)	10% NO	70	11 days−15 y	Mean 65	–	Before elective surgery
Ogawa et al. (19)	Xenon injection	16	5–15 year	92.1 ± 21.5	–	
Chiron et al. (23)	Xe-133 and SPECT	42	2 day−19 year	50–71	5 year	Neurological problems
Takahashi et al. (18)	PET	24	10 day−16 year	–	7 year	Moyamoya, craniosynostosis
Vivalala (22)	TCD	9	12–17	75.2 ± 15.2		Healthy volunteers
Wintermark (24)	CT-perfusion	53	7 day−18 year	40-130-50	2–4 year	TBI, headache, seizure, meningitis, those with normal CT were included
Satterthwaite et al. (29)	MRI-ASL	922	8–22 year	–	<10 year	Healthy
Carsin-Vu et al. (26)	MRI-ASL	84	6 month−15 year	50–69	2–3 year	Headache, seizures
Paniukov et al. (27)	MRI-ASL	96	2–7 year	–	7 year	Healthy

### CBF in Children With Severe TBI

One of the first studies investigating CBF in children with TBI was the study by Kasoff et al. (30) using Xe133 injections in the internal carotid artery. They studied 10 children aged 9 months to 12 years with severe TBI and found hyperemic episodes and poor control of vasomotor tone. Hyperemic episodes were also reported by Bruce et al. who observed cerebral swelling as a common first sign in severe head injured children (31). The study included 85 children and 6 of these underwent CBF measurement showing hyperemia. The authors concluded that the observed cerebral swelling could be explained by acute “vasomotor paralysis” and hyperemia and not by regular brain edema. Therefore, it was suggested that the treatment of high ICP should be focused on the vascular compartment which could be reduced by hyperventilation (4). Also Muizelaar et al. reported hyperemic events in 88% of the TBI children they studied. They concluded that luxury perfusion is common in the pediatric population, although a small group of 6 children was studied (32). These findings were challenged by Skippen et al. who showed that CBF was reduced following TBI in children and that hyperventilation (<4.7 kPa) increased the proportion of children with ischemia (defined as CBF <18 ml/100 g/min) from 28.9 to 73.1%, warranting caution in use of hyperventilation (15).

Several following studies in children with TBI could not find that hyperemia was common, e.g., the study by Sharples et al. (33) who could only detect this in 7% of the children. It should be noted that they used a higher CBF level as normal value, 65 ml/100 g/min.

Using Xenon-CT, Adelson et al. investigated CBF in children <8 years of age with severe TBI (14). The CBF at admission was 25 ml/100 g/min in mean and after 24 h it peaked to 59 ml/100 g/min. The mean CBF during days 2–6 was 55 ml/100 g/min. Children with poor outcome had significantly lower CBF on admission (9.9 ml/100 g/min) compared to those with good outcome (43.9 ml/100 g/min) and several children with admission CBF of <20 ml/100 g/min died.

A subsequent study by Adelson et al. with a larger cohort of 95 children were consistent with these findings (34). The mean admission CBF was 32 ml/100 g/min and there was a significant difference between children who had favorable outcome (46 ml/100 g/min) compared to those that had an unfavorable outcome (18 ml/100 g/min). This important work by Adelson et al. shifted the emphasis away from hyperemia in children with TBI toward the importance of early hypoperfusion and its relation to outcome. The association of initial low CBF following TBI and poor outcome had also been reported earlier by Sharples et al. (33).

In order to maintain adequate CBF cerebral pressure autoregulation is vital and it has been shown that this is disturbed in children suffering TBI (13, 34–37).

Several studies have used different methods and endpoints to assess the cerebral pressure autoregulation in pediatric TBI. Sharples et al. calculated cerebrovascular resistance through CBF measurements (calculated from CVR = CPP/CBF) and preserved cerebral pressure autoregulation was defined as a significant correlation between CPP and CVR (33). Children with worse outcome had impaired cerebral autoregulation defined as above.

Using TCD in 36 severe TBI children, Chaiwat et al. assessed cerebral pressure autoregulation in 36 children with severe TBI by continuous measurements of CPP and flow velocity in MCA during infusion of phenylephrine (37). They report impaired cerebral autoregulation as an independent risk factor for poor 6-month outcome.

Freeman et al. also used TCD and infusion of phenylephrine to assess cerebral pressure autoregulation and reported that children <4 years had a higher incidence of impaired cerebral autoregulation and worse 12-month outcome (38). Adelson et al. used CO_2_ vascular reactivity as a measure of cerebral autoregulation and showed that impaired cerebral autoregulation within 48 h of injury was associated with an unfavorable outcome (34).

In l996 continuous measurement of pressure reactivity index was developed and have been applied to assess cerebral autoregulation (39). Brady et al. evaluate PRx in 21 children with severe TBI and found that non-survivors had impaired cerebral autoregulation (40). This was later also seen by Young et al. in 12 children where PRx showed significant outcome separation between survivors and non-survivors (41). Lewis et al. analyzed the association of PRx and 6 months outcome in 36 children with severe TBI and found worse PRx in patients with unfavorable outcome (42). However, further studies are needed to elucidate if correlation of impaired cerebral autoregulation and outcome in children is causal or just a marker of severity of TBI.

Although the studies above provide valuable information on the injured brain of the pediatric population with TBI there is still a large gap of knowledge (Table 2). As presented above the number of studies assessing CBF in children with severe TBI are limited. The studies cover different age spans ranging 0–18 years and include very few cases they also assess the CBF at different time points post-injury, all of which could hamper a comparison between the studies. In addition, they use different methods to measure CBF and most of them used TCD. Although TCD have advantages such as being non-invasive it is not a direct measurements of CBF and the correlation of flow velocities with actual CBF in the injured brain is weak (43). This calls for further well-designed larger studies in children with severe TBI in order to understand the alterations of CBF post-injury and its correlation to cerebral autoregulation and metabolism and its impact on outcome.

**Table 2:** Summary of papers reporting on CBF measurements and autoregulation in children with severe TBI.

**References**	**Method**	***n***	**Age**	**CBF (ml/100 g/min)/autoregulation**	**CBF measurement time after TBI**	**TBI Severity**
Kasoff et al. (30)	Xe injection	10	9 month−12 year		Hours-2 months	GCS <8
Muizelaar et al. (32)	Xe133 inhalation/inj	32	3–18 year	Good outcome 48.8 ± 24.8	Within 72 h	GCS <7
				Poor outcome 34.7 ± 9.9		
Sharples et al. (33)	10% NO	17	2–16 year	Low CBF in patients with worse outcome	Within 24 h	GCS <8
Skippen et al. (15)	Xe-CT	23	3 month−16 year	Mean 49.6 ± 14.6		GCS <8
Adelson et al. (14)	Xe-CT	30	0–8	Mean 55	Admission—day 9	GCS <8
				Poor outcome 9.9		
				Good outcome 43.9		
Adelson et al. (34)	Xe-CT	95		Mean 32	Admission—day 9	GCS <8
				Good outcome 46		
				Poor outcome 18		
Sharples et al. (13)	10% NO	21	2–16 year	Day 1 40–60 ml/100 g/ml	6–57 h	GCS <8
Muizelaar et al. (35)	Xe133-phenylephrine	26	8–18 year	No correlation between autoregulation and outcome	Before and after 36 h	GCS <7
Vivalala (36)	TCD	28	5–15 year	Impaired autoregulation-worse 6 m outcome	Within 72 h	GCS <9
Freeman et al. (38)	TCD-phenylephrine	37	8 month−16	Impaired autoregulation in <4 year	Within 72 h	GCS <13
Chaiwat et al. (37)	TCD	36	8 month−16 year	Impaired autoregulation-worse outcome	Within 72 h	GCS <9
Brady et al. (40)	PRx	21	3 month−15 year	PRx associated with survival		GCS <8
Young et al. (41)	PRx	12	3 month−13 year	PRx correlates with 6 months outcome		GCS <8
Lewis et al. (42)	PRx	36	6 month−16 year	PRx correlates with outcome		GCS <8

## Conclusion

It is apparent that the pathophysiology of brain injury changes from birth to 18 years of age. Children's physiology and anatomy changes tremendously during growth and during the development of the brain regarding e.g., skull dimensions, suture elasticity, vascular maturation, tortuosity, gray, and white matter ratio (44). This makes it very likely that not only the biomechanics of injury to the brain is different but also the response of the brain to the injury. The differences in CBF found in healthy children of different ages, as presented in this review, suggest that the tolerability/vulnerability of the brain is very different depending on the age and that the differences may be very large even with very small differences in age.

Accordingly, the outcome following TBI has shown to be age depended with children under the age of 4–5 years having worse outcome. More studies of specific cerebral hemodynamic changes following TBI in different age groups of children are needed in order to improve the management of children with severe TBI. The importance of this is underlined by estimates from the U.S. Centers for Disease Prevention and Control that show rates of TBI-related emergency department visits for children 0–4 years increased by more than 50%.

## Author Contributions

ER conceptualized the idea and wrote the paper. PN provided critical feedback. PE provided with critical feedback and helped to shape the manuscript.

### Conflict of Interest

The authors declare that the research was conducted in the absence of any commercial or financial relationships that could be construed as a potential conflict of interest.
